# Supplementary material for: A bipolar disorder-associated ultra-rare AKAP11 protein-truncating variant attenuates stimulus-dependent PKA activation and induces anxiety- and depression-related behaviors in mice
Source: bioRxiv. 2026 Sep 28:2026.09.19.752887. Preprint. [Version 2] doi: 10.64898/2026.09.19.752887 (PMC13648366; doi:10.64898/2026.09.19.752887)
Supplement: Supplementary file 1 [file NIHPP2026.09.19.752887v2-supplement-9.pdf]

bioRxiv preprint doi: https://doi.org/10.64898/2026.09.19.752887; this version posted September 28, 2026. The copyright holder for this preprint (which was not certified by peer review) is the author/funder, who has granted bioRxiv a license to display the preprint in perpetuity. It is made available under a CC-BY 4.0 International license.

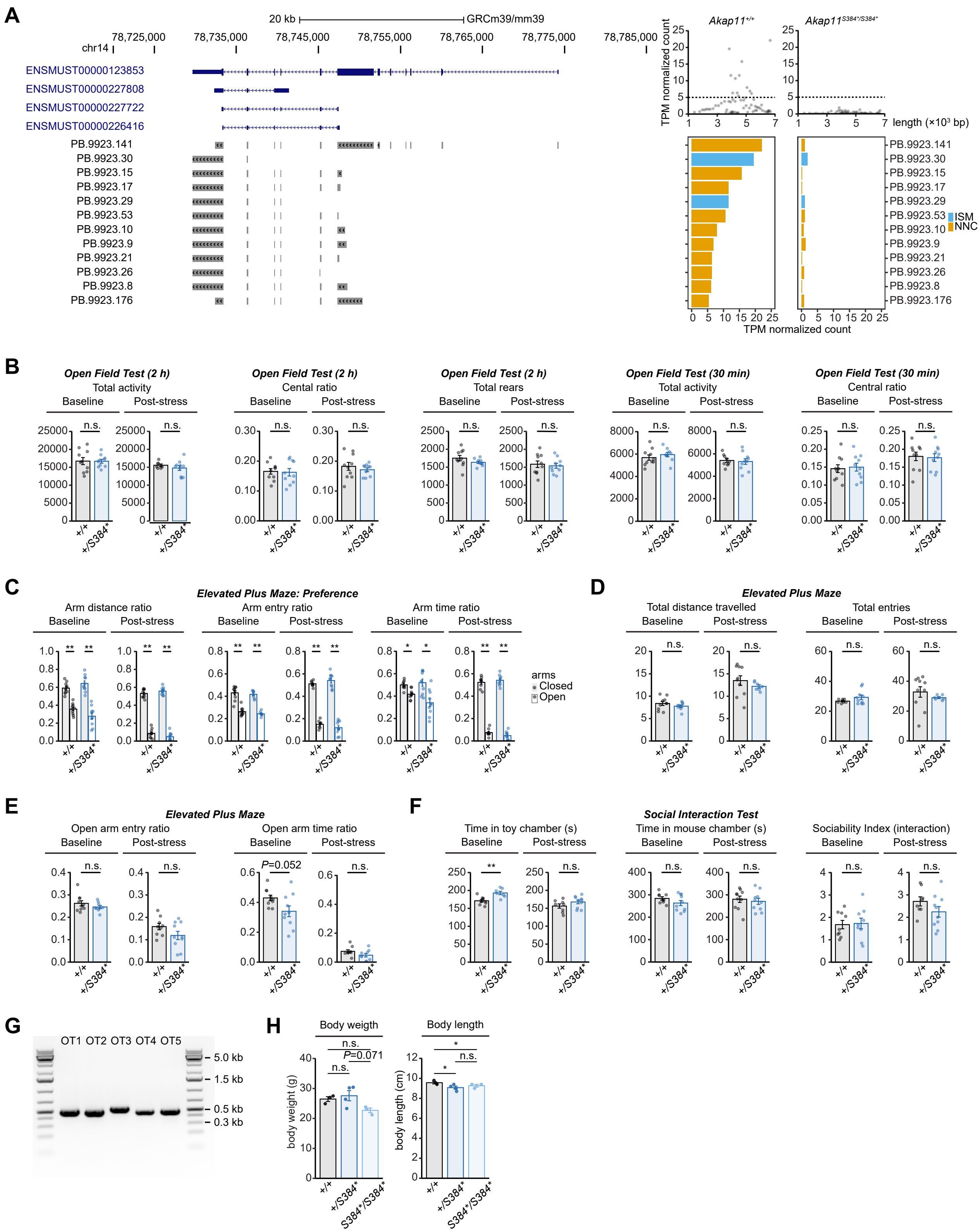

Supplementary Fig. 1. *Akap11* mRNA isoforms in homozygous mutant mice, and behavioral characterization of heterozygous mutant mice. Related to Fig. 1.

bioRxiv preprint doi: https://doi.org/10.64898/2026.09.19.752887; this version posted September 28, 2026. The copyright holder for this preprint (which was not certified by peer review) is the author/funder, who has granted bioRxiv a license to display the preprint in perpetuity. It is made available under a CC-BY 4.0 International license.

A. Genomic tracks showing four annotated *Akap11* transcript variants (blue) and *Akap11* isoforms with transcripts per million (TPM) ≥ 5 detected in the *Akap11*$^{+/+}$ cortical sample (gray) on the GRCm39/mm39 genome assembly. Upper right, *Akap11* isoform expression level, shown as TPM-normalized count, plotted against isoform length in *Akap11*$^{+/+}$ and *Akap11*$^{+/S384*}$ samples. Lower right, bar plots showing TPM-normalized expression levels for the isoforms displayed in the genomic tracks. ISM, incomplete splice matches, NNC, novel not in catalog.

B. *Akap11*$^{+/S384*}$ mice showed normal total activity, central ratio, and total rears in 2-h open field test at baseline and after three weeks of isolated housing. No difference was detected between genotypes in total activity or central ratio during the first 30 min of the open field test. n.s. $P >$ 0.05 via Mann-Whitney U test. Outlier removal and full statistical results are described in Supplementary Table 2A.

C. Both *Akap11*$^{+/+}$ and *Akap11*$^{+/S384*}$ mice showed a preference for the closed arms in the elevated plus maze test, as indicated by the distance, entry and time measures. **$P \leq 0.01$; *$P \leq$ 0.05 via paired Wilcoxon signed-rank test. Outlier removal and full statistical results are provided in Supplementary Table 2B.

D. *Akap11*$^{+/+}$ and *Akap11*$^{+/S384*}$ mice did not differ in the total activity level in the elevated plus maze, measured by total distance travelled and total zone entries. n.s. $P > 0.05$ via Mann-Whitney U test. Outlier removal and full statistical results are provided in Supplementary Table 2B.

E. *Akap11*$^{+/S384*}$ mice showed normal open-arm entry ratio in the elevated plus maze test at baseline and after isolated housing. Open-arm time ratio showed a trend toward reduction in *Akap11*$^{+/S384*}$ mice at baseline ($P = 0.052$) which was not observed after isolated housing. n.s. $P > 0.05$ via Mann-Whitney U test. Outlier removal and full statistical results are provided in Supplementary Table 2B.

F. In the social interaction test, *Akap11*$^{+/S384*}$ mice spent significantly more time in the toy chamber than the control mice at baseline ($P = 0.0081$), but not after isolated housing. Time spent in the mouse chamber and interaction-based sociability index did not differ between genotypes before or after isolated housing. **$P \leq 0.01$; n.s., $P > 0.05$ via Mann-Whitney U test. Outlier removal and full statistical results are provided in Supplementary Table 2C.

G. Polymerase chain reaction (PCR) amplification products for the top five predicted sgRNA off-target sites (OT1-5).

H. Bar plots showing effect of genotype on body weight (left) and body length (right) of 12-week-old male mice. Genotype had a marginal effect on body weight ($P = 0.065$ via a rank-transformed linear model with genotype and litter as fixed effects). *Post-hoc* analysis with Tukey's test showed that homozygous mutant mice had marginally lower body weight than heterozygous mutant mice ($P = 0.071$), and no difference between other genotype pairs. Genotype significantly affected body length ($P = 0.0035$ via rank-transformed linear model with genotype and litter as fixed effects). Both heterozygous and homozygous mutants were shorter than control mice ($P = 0.014$ and $P = 0.028$, respectively, via Tukey's test). No difference

bioRxiv preprint doi: https://doi.org/10.64898/2026.09.19.752887; this version posted September 28, 2026. The copyright holder for this preprint (which was not certified by peer review) is the author/funder, who has granted bioRxiv a license to display the preprint in perpetuity. It is made available under a CC-BY 4.0 International license.

between the heterozygous and homozygous mutants was observed. *$P \leq 0.05$; n.s. $P > 0.05$ via Tukey's test.

bioRxiv preprint doi: https://doi.org/10.64898/2026.09.19.752887; this version posted September 28, 2026. The copyright holder for this preprint (which was not certified by peer review) is the author/funder, who has granted bioRxiv a license to display the preprint in perpetuity. It is made available under a CC-BY 4.0 International license.

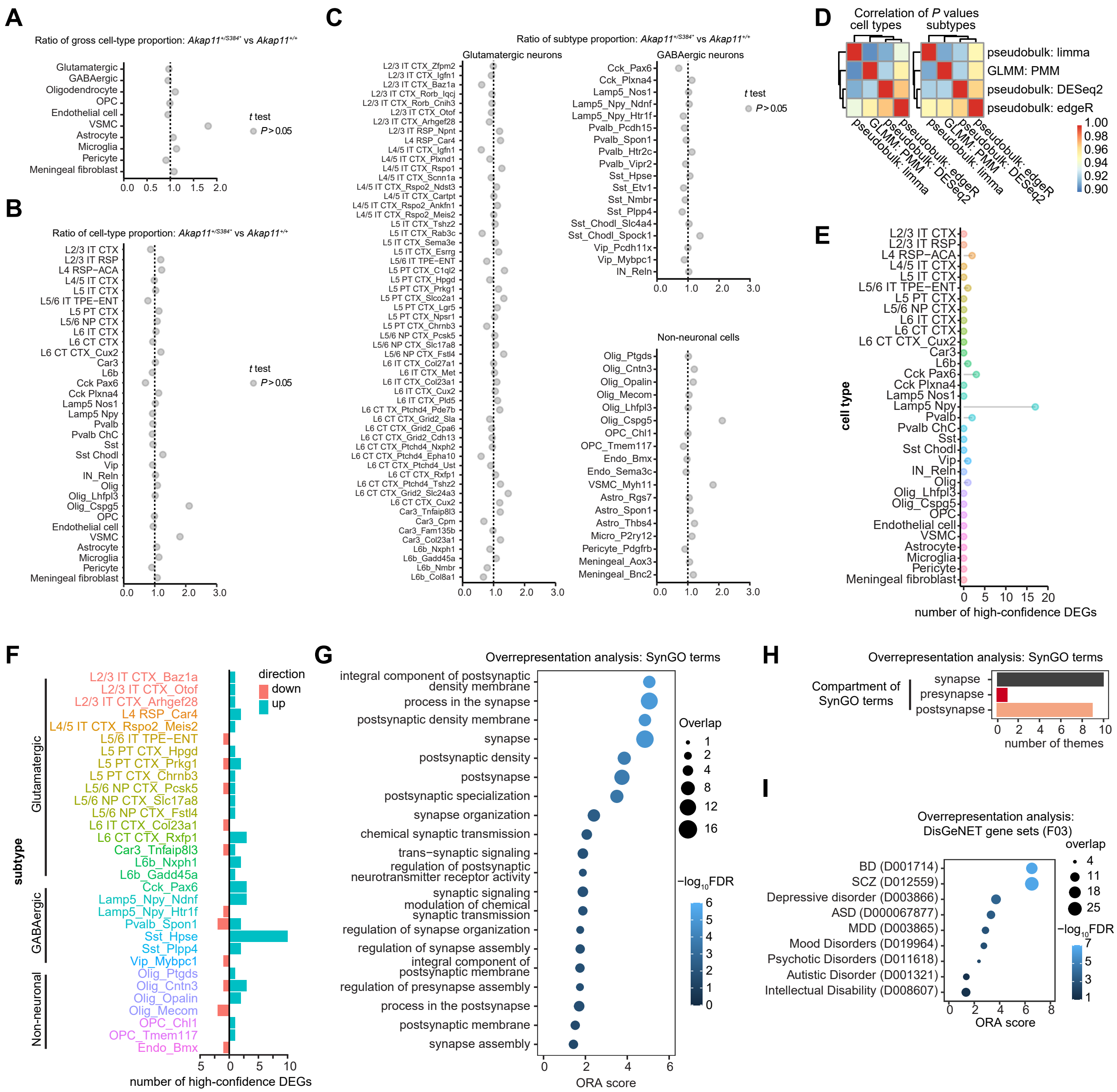

Supplementary Fig. 2. Single-nucleus RNA sequencing profiling of the neocortex of heterozygous mutant mice. Related to Fig. 2.

A. Ratio of broad cell type proportions between genotypes, calculated as *Akap11*$^{+/S384*}$ over *Akap11*$^{+/+}$. Gray dots, $P > 0.05$ in cell type composition test via moderated *t*-test using *propeller*.

B. Ratio of cell type proportions between genotypes, calculated as *Akap11*$^{+/S384*}$ over *Akap11*$^{+/+}$. Gray dots, $P > 0.05$ in cell type composition test via moderated *t*-test using *propeller*.

C. Ratio of subtype proportions between genotypes, calculated as *Akap11*$^{+/S384*}$ over *Akap11*$^{+/+}$. Gray dots, $P > 0.05$ in cell type composition test via moderated *t*-test using *propeller*.

D. Heatmap showing Pearson correlations among *P* values from three pseudobulk differential expression (DE) tests (via *edgeR*, *DESeq2*, and *limma*) and a single-cell Poisson-gamma mixed

bioRxiv preprint doi: https://doi.org/10.64898/2026.09.19.752887; this version posted September 28, 2026. The copyright holder for this preprint (which was not certified by peer review) is the author/funder, who has granted bioRxiv a license to display the preprint in perpetuity. It is made available under a CC-BY 4.0 International license.

model (PMM)-based DE test (via *nebula*). Correlations are shown for the 33 cell types (left) and the 92 subtypes (right).

E. Lollipop plot showing the number of high-confidence differentially expressed genes (DEGs) detected in each of the 33 cell types. High-confidence DEGs were defined as genes with FDR ≤ 0.10 in pseudobulk DE test via quasi-likelihood negative binomial generalized log-linear model with *edgeR*, and nominal $P \leq 0.05$ in PMM-based single-cell DE test, with concordant direction of change between methods.

F. Number of high-confidence DEGs detected in each subtype. Green indicates upregulated genes, and red indicates downregulated genes in $Akap11^{+/S384*}$ mice. Subtype labels are colored according to the cell type to which they belong.

G. Synapse-specific gene ontology (SynGO) terms overrepresented among upregulated high-confidence DEGs across all subtypes. Significant overrepresentation was defined as FDR ≤ 0.05 via hypergeometric test. The overrepresentation analysis (ORA) score was calculated as $-\log_{10}$FDR multiplied by the direction of change, with positive values indicating enrichment among genes upregulated in $Akap11^{+/S384*}$ mice, and negative values indicating enrichment among downregulated genes.

H. Distribution of overrepresented SynGO terms by synaptic compartment.

I. DisGeNET gene sets in the MeSH F03 category (“mental disorders”) overrepresented among the extended high-confidence DEGs. Significant overrepresentation was defined as FDR ≤ 0.05 via hypergeometric test. The ORA score was calculated as $-\log_{10}$FDR multiplied by the direction of change. Abbreviations: ASD=autism spectrum disorder, BD=bipolar disorder, MDD=major depressive disorder, and SCZ=schizophrenia.

bioRxiv preprint doi: https://doi.org/10.64898/2026.09.19.752887; this version posted September 28, 2026. The copyright holder for this preprint (which was not certified by peer review) is the author/funder, who has granted bioRxiv a license to display the preprint in perpetuity. It is made available under a CC-BY 4.0 International license.

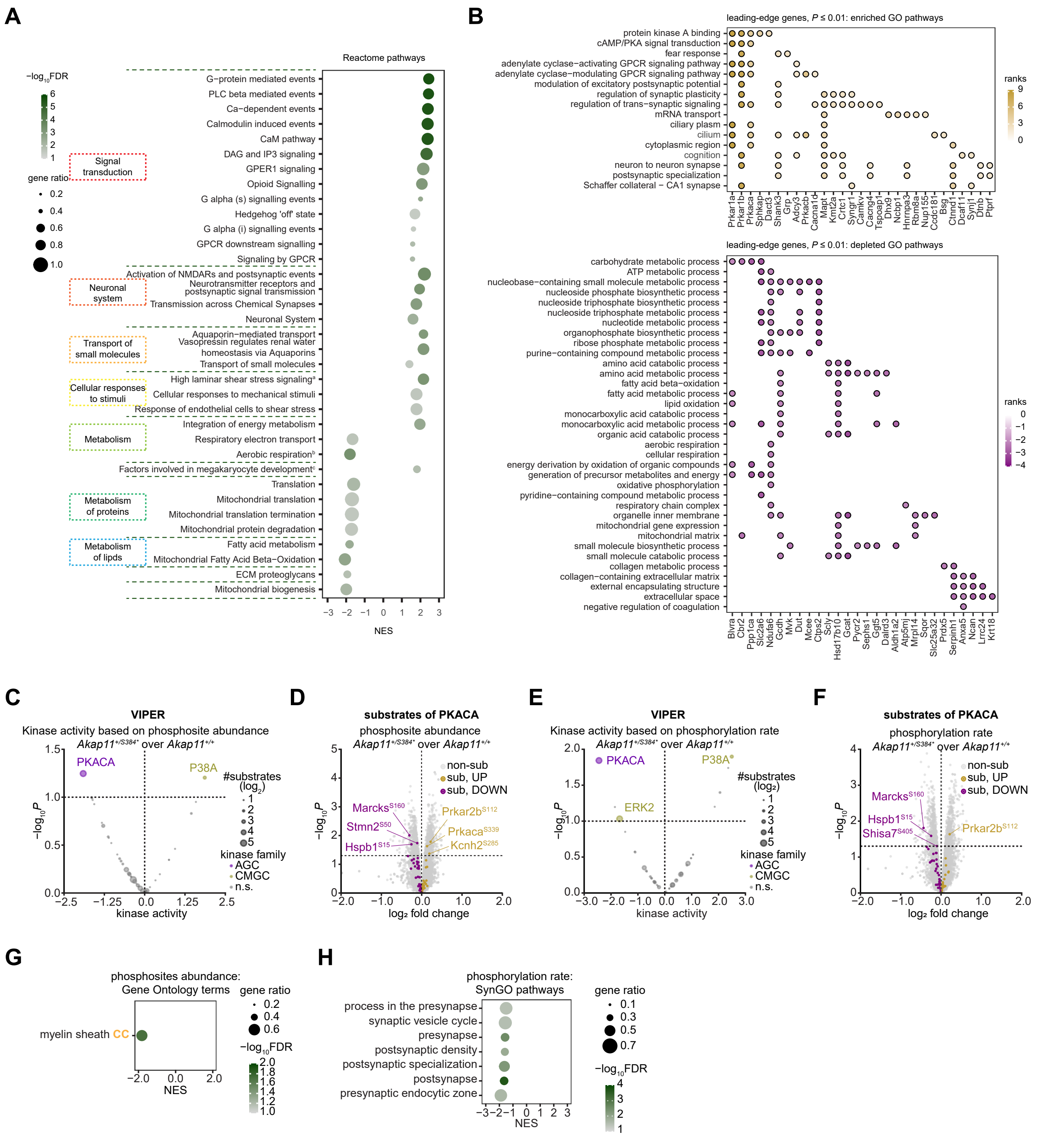

Supplementary Fig. 3. Proteomic profiling of the cortex of heterozygous mutant mice. Related to Fig. 4.

A. Reactome pathways significantly enriched or depleted in *Akap11*$^{+/S384*}$ mice via permutation test. Positive and negative normalized enrichment scores (NES) indicate enrichment and depletion, respectively in the heterozygous mice. Gene ratio was computed as the proportion of pathway genes represented in the ranked protein list. Significance was defined as FDR ≤ 0.05. Pathways are grouped into 10 groups according to the Reactome pathway hierarchy (retrieved

bioRxiv preprint doi: https://doi.org/10.64898/2026.09.19.752887; this version posted September 28, 2026. The copyright holder for this preprint (which was not certified by peer review) is the author/funder, who has granted bioRxiv a license to display the preprint in perpetuity. It is made available under a CC-BY 4.0 International license.

from https://reactome.org/). Semantic summary are shown for categories containing multiple pathways. Truncated labels: [a]High laminar flow shear stress activates signaling by PIEZO1 and PECAM1:CDH5:KDR in endothelial cells; [b]Aerobic respiration and respiratory electron transport; [c]Factors involved in megakaryocyte development and platelet production.

B. Leading-edge proteins for the enriched (upper) and depleted (lower) Gene Ontology (GO) terms shown in Fig. 4C. Color indicates protein rank, which was computed as $-\log_{10}P$ x sign ($\log_2$ fold change) from the differential protein abundance analysis. Positive and negative ranks indicate increased and decreased abundance, respectively, in *Akap11*$^{+/S384*}$ mice. Only leading-edge proteins with $P \leq 0.01$ and GO terms with at least one such protein are shown.

C, E. Volcano plots showing predicted kinase activity inferred from phosphosite abundance (C) or phosphorylation rate (E), using an alternative test method (VIPER). VIPER was applied to $-\log_{10}P$ x sign($\log_2$ fold change). Positive and negative kinase activity scores indicate increased and decreased kinase activity, respectively, in *Akap11*$^{+/S384*}$ mice. Kinases were considered significant at $P \leq 0.05$ with at least three substrates present.

D, F. Volcano plots showing genotype-dependent differences in phosphosite abundance (D), and phosphorylation rate (F), highlighting the predicted PKACA substrates associated with the *RoKAI* test. Positive and negative $\log_2$ fold changes indicate increased and decreased phosphosite abundance or phosphorylation rate, respectively, in *Akap11*$^{+/S384*}$ mice. Horizontal and vertical dashed lines indicate P at 0.05 and $\log_2$ fold change at 0, respectively.

G. Gene set enrichment analysis (GSEA) of GO terms using phosphosites ranked $-\log_{10}P$ x sign($\log_2$ fold change), based on phosphosite abundance. Positive and negative NES values indicate pathways associated with proteins harboring increased or decreased phosphosite abundance, respectively, in *Akap11*$^{+/S384*}$ mice. P values were estimated via permutation test, and significance was defined as FDR ≤ 0.05. Gene ratio was computed as the proportion of pathway genes present in the phosphosite dataset.

H. GSEA of SynGO terms using phosphosites ranked by $-\log_{10}P$ x sign($\log_2$ fold change) based on phosphorylation rate. Positive and negative NES values indicate SynGO pathways associated with proteins harboring increased or decreased phosphorylation rates, respectively, in *Akap11*$^{+/S384*}$ mice.

bioRxiv preprint doi: https://doi.org/10.64898/2026.09.19.752887; this version posted September 28, 2026. The copyright holder for this preprint (which was not certified by peer review) is the author/funder, who has granted bioRxiv a license to display the preprint in perpetuity. It is made available under a CC-BY 4.0 International license.

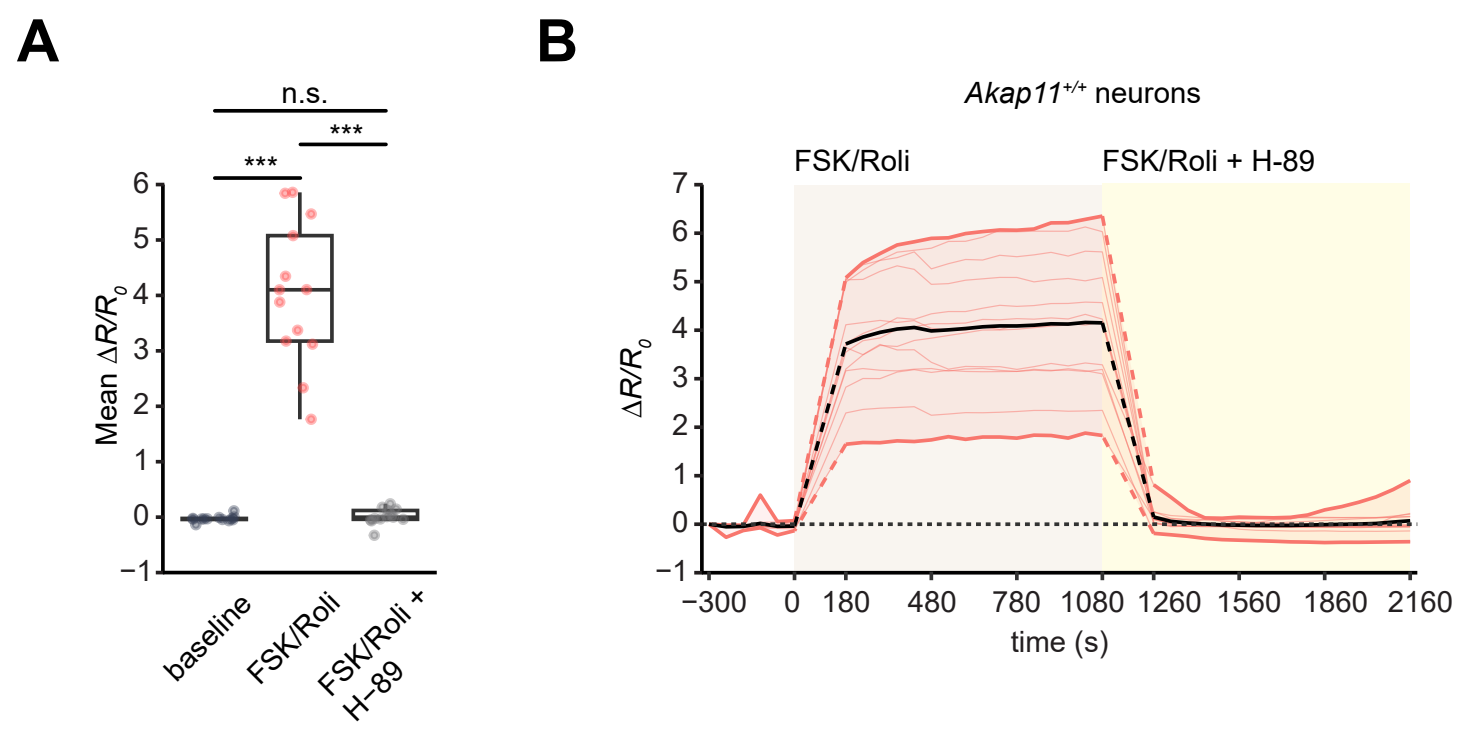

Supplementary Fig. 4. Validation of PKA sensor. Related to Fig. 5.

A. Boxplots showing the mean change in F_{488}/F_{405} ratio normalized to the first time point (mean $\Delta R/R_0$) in *Akap11*$^{+/+}$ neurons at baseline, in response to forskolin/rolipram (FSK/Roli), and in response to addition of H-89 in the continued presence of forskolin/rolipram (FSK/Roli+H-89). Forskolin/rolipram significantly increased sensor response relative to baseline (FDR = 7.32 x 10^{-4}), whereas subsequent addition of H-89 restored the response to baseline (FSK/Roli+H-89 vs. FSK/Roli: FDR = 7.32 x 10^{-4}; FSK/Roli+H-89 vs. baseline: FDR = 0.50). N = 2 embryos, n= 13 neurons. ***FDR ≤ 0.001, n.s., FDR > 0.05 via paired Wilcoxon signed-rank test. For boxplots, the bottom edge, midline, and top edge represent the first quantile, median, and third quantile, respectively. Whiskers represent 1.5 x interquartile range from the box edges. Full statistical results are shown in Supplementary Table 7B.

B. Time course of change in $\Delta R/R_0$ for the neurons shown in A. Dashed lines indicate intervals during drug application, when imaging was paused.
